# Supplementary material for: Ni Oxidation State and Ligand Saturation Impact on the Capability of Octaazamacrocyclic Complexes to Bind and Reduce CO2
Source: Molecules. 2021 Jul 7;26(14):4139. doi: 10.3390/molecules26144139 (PMC8307626; doi:10.3390/molecules26144139)
Supplement: Supplementary file 1 [file molecules-26-04139-s001.zip › SI_mdpi_NiL_CO2_affinity_revision2_final.pdf]

Ni oxidation state and ligand saturation impact on the capability of octaazamacrocyclic complexes to bind and reduce CO_2

Supplementary Materials

Barbora Vénosová,[1] Ingrid Jelemenská,[1,2] Jozef Kožíšek,[1] Peter Rapta,[1] Michal Zalibera,[1] Michal Novotný,[3] Vladimir B. Arion,[4] Lukáš Bučinský[1,*]

[1]Institute of Physical Chemistry and Chemical Physics, Faculty of Chemical and Food Technology, Slovak University of Technology in Bratislava, Radlinského 9, SK-81237 Bratislava, Slovak Republic

[2]Department of Chemistry, Faculty of Natural Sciences, Constantine the Philosopher University in Nitra, 949 74 Nitra, Slovak Republic

[3]Department of Physics, Faculty of Science, University of Ostrava, 30.dubna 22, 701 03 Ostrava, Czech Republic

[4]University of Vienna, Institute of Inorganic Chemistry, Währinger Strasse 42, A-1090 Vienna, Austria

[*]lukas.bucinsky@stuba.sk

Content

Table S1. Total B3LYP/6-311G* energies/enthalpies of different species studied 2

Table S2. Selected B3LYP/6-311G* bond distances (Å) and angles (°) of a) [**NiL**] and b) [**NiLH**] 3

Table S3. B3LYP/6-311G* MPA charges of a) [**NiL**] and b) [**NiLH**] 4

Table S4. B3LYP/6-311G* QTAIM charges of a) [**NiL**] and b) [**NiLH**] 5

Table S5. B3LYP/6-311G* QTAIM BCP characteristics of a) [**NiL**] and b) [**NiLH**] 6

Table S6. B3LYP/6-311G* MPA of a) [**NiL**] and b) [**NiLH**] localized molecular orbitals on Ni 8

Figure S1. Comparison of geometries of complexes studied without and with CO_2 10

Figure S2. Frontier orbitals of a) ^{1}[**NiL**]^0 and ^{3}[**NiL**]$^{2-}$; b) ^{1}[**NiLH**]^0 and ^{2}[**NiLH**]$^+$ 11

Figure S3. Frontier orbitals of a) ^{1}[**NiL-CO$_2$**]^0 and ^{3}[**NiL-CO$_2$**]$^{2-}$; b) ^{1}[**NiLH-CO$_2$**]^0 and ^{2}[**NiLH-CO$_2$**]$^+$ 13

Table S1. Total B3LYP/6-311G* energies/enthalpies of different [**NiL**], [**NiL-CO$_2$**], [**NiLH**], [**NiLH-CO$_2$**] species studied and CO_2 (all values are in A.U.). [**NiLH$_X$**] species denote geometries, where different R and S conformations were tested for the given atom(s) X (for atom labels see Figure 1 in the manuscript).

	E	H		E	H
^{3}[**NiL**]$^{2-}$	-3250.503603	-3250.142369	^{3}[**NiL-CO$_2$**]$^{2-}$	-3439.157621	-3438.780621
^{1}[**NiL**]$^{2-}$	-3250.510626	-3250.118574	^{1}[**NiL-CO$_2$**]$^{2-}$	-3439.157234	-3438.778617
^{2}[**NiL**]$^{-}$	-3250.466015	-3250.101912	^{2}[**NiL-CO$_2$**]$^{-}$	-3439.111696	-3438.730981
^{1}[**NiL**]^{0}	-3250.378540	-3250.011645	^{1}[**NiL-CO$_2$**]^{0}	-3439.023880	-3438.640248
^{2}[**NiL**]$^{+}$	-3250.186230	-3249.819337	^{2}[**NiL-CO$_2$**]$^{+}$	-3438.830920	-3438.448238
^{1}[**NiL**]$^{2+}$	-3249.948182	-3249.580637	^{1}[**NiL-CO$_2$**]$^{2+}$	-3438.592560	-3438.208037
^{1}[**NiLH**]^{0}	-3258.616497	-3258.079627	^{1}[**NiLH-CO$_2$**]^{0}	-3447.321361	-3446.765337
^{2}[**NiLH**]$^{+}$	-3258.578456	-3258.036079	^{2}[**NiLH-CO$_2$**]$^{+}$	-3447.22514	-3446.665791
^{2}[**NiLH$_{C1}$**]$^{+}$	-3258.574194	-3258.031725	^{2}[**NiLH$_{C1}$-CO$_2$**]$^{+}$	-3447.221017	-3446.661914
^{2}[**NiLH$_{C5}$**]$^{+}$	-3258.577090	-3258.034923	^{2}[**NiLH$_{C5}$-CO$_2$**]$^{+}$	-3447.224161	-3446.664701
^{1}[**NiLH**]$^{2+}$	-3258.432714	-3257.886345		-	-
^{1}[**NiLH$_{C1}$**]$^{2+}$	-3258.428449	-3257.881841			
^{1}[**NiLH $_{C3}$**]$^{2+}$	-3258.430081	-3257.883263			
^{1}[**NiLH $_{C5}$**]$^{2+}$	-3258.430207	-3257.882894			
^{1}[**NiLH $_{C1C3}$**]$^{2+}$	-3258.422065	-3257.875684			
**CO_2**	-188.643673	-188.628479	^{1}[**NiL$_{fr}$CO$_2$**]^{0}	-3438.976674	-3438.542170

Table S2a. Selected B3LYP/6-311G* bond distances (Å) and angles (°) of [**NiL**] complexes (according to the notation in Figure 1).

**Distances**	^{3}[**NiL**]$^{2-}$	^{3}[**NiL-CO$_2$**]$^{2-}$	^{1}[**NiL**]$^{2-}$	^{2}[**NiL**]$^-$	^{1}[**NiL**]^0	^{1}[**NiL-CO$_2$**]^0	^{1}[**NiL$_{fr}$CO$_2$**]^0	^{2}[**NiL**]$^+$	^{1}[**NiL**]$^{2+}$
**Ni-N(1)**	2.01	1.99	1.92	1.91	1.90	1.90	1.89	1.91	1.92
**Ni-N(3)**	1.97	2.05	1.90	1.91	1.91	1.91	1.95	1.91	1.90
**Ni-N(5)**	1.94	1.95	1.84	1.85	1.83	1.83	1.84	1.83	1.84
**Ni-N(7)**	1.92	1.95	1.87	1.85	1.86	1.86	1.89	1.86	1.87
**Angles**									
**N(1)NiN(3)**	106.70	106.25	102.95	102.37	102.74	102.74	102.96	102.87	102.95
**N(3)NiN(5)**	80.67	78.96	82.75	82.41	82.10	82.10	81.19	82.42	82.75
**N(5)NiN(7)**	91.08	91.69	92.60	91.85	92.18	92.18	92.49	92.56	92.60
**N(7)NiN(1)**	81.38	80.89	81.94	83.30	83.15	83.15	82.68	82.48	81.94
**N(1)NiN(5)**	168.53	170.39	169.35	171.17	171.67	171.67	175.00	170.92	169.35
**N(3)NiN(7)**	171.73	160.68	174.97	174.25	174.02	174.02	160.48	174.22	174.97
**NiN(1)N(2)**	132.28	129.05	132.44	132.89	133.20	133.20	130.18	132.41	132.44
**NiN(3)N(4)**	114.30	112.99	112.99	113.93	113.94	113.94	113.13	113.57	112.99
**NiN(5)N(6)**	130.56	129.69	131.03	131.72	131.26	131.26	130.24	131.05	131.03
**NiN(7)N(8)**	116.17	115.34	117.02	115.97	115.77	115.77	115.14	116.42	117.02
**NiN(3)C(3)**	124.21	124.91	129.67	129.18	129.26	129.26	129.31	129.48	129.67
**NiN(7)C(6)**	126.34	126.49	126.41	127.20	127.46	127.46	126.95	126.94	126.41
**CO$_2$^{a}**	-	137.62	-	-	-	179.53	145.24	-	-
**NiCO^{b,c}**	-	111.00	-	-	-	87.90	105.56	-	-
**N(3)NiC^b**	-	94.83	-	-	-	82.70	99.51	-	-
**N(7)NiC^b**	-	102.87	-	-			99.51	-	-

^{a} O-C-O angle in CO_2; ^{b} C denotes the carbon in CO_2; ^{c} O denotes one of the oxygens in CO_2

Table S2b. Selected B3LYP/6-311G* bond distances (Å) and angles (°) of [**NiLH**] (according to the notation in Figure 1).

**Distances**	^{1}[**NiLH**]^0	^{1}[**NiLH-CO$_2$**]^0	^{2}[**NiLH**]$^+$	^{2}[**NiLH-CO$_2$**]$^+$	^{1}[**NiLH**]$^{2+}$
**Ni-N(1)**	2.96	3.08	2.10	2.11	1.95
**Ni-N(3)**	1.91	2.00	2.06	2.09	1.95
**Ni-N(5)**	2.77	2.22	2.06	2.06	1.92
**Ni-N(7)**	1.88	2.01	2.05	2.08	1.96
**Angles**					
**N(1)NiN(3)**	114.30	106.85	100.59	101.08	97.28
**N(3)NiN(5)**	73.47	80.03	84.14	83.21	86.89
**N(5)NiN(7)**	100.24	101.33	89.57	89.97	89.25
**N(7)NiN(1)**	71.77	70.55	85.14	83.38	86.50
**N(1)NiN(5)**	172.00	149.29	172.87	167.37	175.01
**N(3)NiN(7)**	168.09	170.22	171.30	168.17	175.84
**NiN(1)N(2)**	107.62	113.70	110.77	109.97	107.80
**NiN(3)N(4)**	116.42	108.66	103.22	103.93	109.90
**NiN(5)N(6)**	94.11	119.89	108.97	110.76	111.79
**NiN(7)N(8)**	119.95	118.78	108.19	108.96	109.69
**NiN(3)C(3)**	119.55	120.72	113.29	114.18	117.35
**NiN(7)C(6)**	114.16	116.28	114.85	116.65	114.23
**CO$_2$^{a}**	-	129.12	-	146.63	-
**NiCO^{b,c}**	-	118.53	-	105.26	-
**N(3)NiC^b**	-	94.55	-	93.88	-
**N(7)NiC^b**	-	94.11	-	96.30	-

^{a} O-C-O angle in CO_2; ^{b} C denotes the carbon in CO_2; ^{c} O denotes one of the oxygens in CO_2

Table S3a. B3LYP/6-311G* MPA charges of [**NiL**] complexes.

Atoms	^{3}[NiL]$^{2-}$	^{3}[NiL-CO_2]$^{2-}$	^{1}[NiL]$^{2-}$	^{2}[NiL]$^{2-}$	^{1}[NiL]^{0}	^{1}[NiL-CO_2]^{0}	^{1}[$NiL_{fr}CO_2$]^{0}	^{2}[NiL]$^{+}$	^{1}[NiL]$^{2+}$
**Ni**	0.730	1.059	0.887	0.958	1.003	1.002	1.102	1.047	1.096
**S(1)**	-0.022	0.006	-0.010	0.057	0.126	0.127	0.152	0.231	0.339
**S(2)**	-0.034	0.001	-0.020	0.064	0.146	0.147	0.171	0.219	0.290
**N(1)**	-0.522	-0.521	-0.556	-0.528	-0.465	-0.464	-0.442	-0.444	-0.388
**N(2)**	-0.509	-0.516	-0.525	-0.531	-0.528	-0.529	-0.533	-0.477	-0.450
**N(3)**	-0.478	-0.469	-0.458	-0.482	-0.494	-0.494	-0.48	-0.419	-0.340
**N(4)**	-0.428	-0.402	-0.436	-0.434	-0.433	-0.432	-0.412	-0.437	-0.439
**N(5)**	-0.358	-0.345	-0.355	-0.330	-0.300	-0.297	-0.274	-0.241	-0.193
**N(6)**	-0.323	-0.311	-0.372	-0.291	-0.212	-0.211	-0.196	-0.177	-0.117
**N(7)**	-0.310	-0.301	-0.279	-0.290	-0.299	-0.296	-0.278	-0.210	-0.122
**N(8)**	-0.231	-0.216	-0.232	-0.216	-0.203	-0.204	-0.204	-0.189	-0.175
**C(1)**	0.107	0.114	0.109	0.121	0.150	0.151	0.154	-0.725	-0.714
**C(2)**	-0.586	-0.610	-0.595	-0.615	-0.627	-0.629	-0.626	-0.636	-0.643
**C(3)**	0.207	0.252	0.225	0.238	0.257	0.257	0.269	0.279	0.296
**C(4)**	0.273	0.323	0.278	0.339	0.409	0.410	0.422	0.430	0.452
**C(5)**	0.228	0.230	0.199	0.259	0.295	0.291	0.283	0.294	0.287
**C(6)**	0.211	0.215	0.183	0.244	0.289	0.286	0.289	0.306	0.313
**C(7)**	0.205	0.239	0.175	0.256	0.319	0.317	0.344	0.344	0.363
**O(1)**		-0.417				-0.260	-0.324		
**C**		0.109				0.515	0.216		
**O(2)**		-0.408				-0.260	-0.321		

Table S3b. B3LYP/6-311G* MPA charges of [**NiLH**]complexes.

Atoms	^{1}[NiLH]^{0}	^{1}[NiLH-CO_2]^{0}	^{2}[NiLH]$^{+}$	^{2}[NiLH-CO_2]$^{+}$	^{1}[NiLH]$^{2+}$	CO_2
**Ni**	0.038	0.840	0.761	1.148	1.049	
**S(1)**	0.001	0.033	0.096	0.123	0.166	
**S(2)**	-0.004	0.056	0.096	0.125	0.177	
**N(1)**	-0.582	-0.629	-0.591	-0.613	-0.608	
**N(2)**	-0.419	-0.551	-0.565	-0.579	-0.591	
**N(3)**	-0.569	-0.408	-0.580	-0.602	-0.604	
**N(4)**	-0.408	-0.604	-0.616	-0.639	-0.629	
**N(5)**	-0.453	-0.420	-0.420	-0.416	-0.401	
**N(6)**	-0.399	-0.427	-0.389	-0.387	-0.358	
**N(7)**	-0.405	-0.396	-0.378	-0.364	-0.351	
**N(8)**	-0.613	-0.628	-0.667	-0.665	-0.680	
**C(1)**	-0.673	-0.650	-0.125	-0.129	-0.151	
**C(2)**	-0.060	-0.107	-0.423	-0.421	-0.411	
**C(3)**	-0.450	-0.445	-0.633	-0.631	-0.624	
**C(4)**	-0.588	-0.639	-0.441	-0.446	-0.427	
**C(5)**	-0.408	-0.432	-0.776	-0.781	-0.786	
**C(6)**	-0.780	-0.785	-0.085	-0.088	-0.111	
**C(7)**	-0.063	-0.061	-0.021	-0.034	-0.059	
**O(1)**		-0.478		-0.328		-0.254
**C**		0.033		0.189		0.501
**O(2)**		-0.482		-0.327		-0.254

Table S4a. B3LYP/6-311G* QTAIM charges of [**NiL**] complexes.

Atoms	^{3}[NiL]$^{2-}$	^{3}[NiL-CO$_2$]$^{2-}$	^{1}[NiL]$^{2-}$	^{2}[NiL]$^{-}$	^{1}[NiL]^{0}	^{1}[NiL-CO$_2$]^{0}	^{1}[NiL$_{fr}$CO$_2$]^{0}	^{2}[NiL]$^{+}$	^{1}[NiL]$^{2+}$
**Ni**	0.741	0.943	0.89	0.942	0.967	0.965	1.000	0.995	1.029
**S(1)**	-0.023	-0.027	-0.04	0.026	0.094	0.095	0.120	0.203	0.321
**S(2)**	-0.011	-0.020	-0.03	0.045	0.118	0.120	0.141	0.192	0.270
**N(1)**	-0.817	-0.759	-0.74	-0.761	-0.772	-0.770	-0.751	-0.679	-0.541
**N(2)**	-0.762	-0.750	-0.74	-0.748	-0.763	-0.763	-0.728	-0.750	-0.737
**N(3)**	-0.692	-0.771	-0.79	-0.747	-0.683	-0.684	-0.663	-0.568	-0.447
**N(4)**	-0.827	-0.803	-0.79	-0.806	-0.824	-0.822	-0.804	-0.810	-0.795
**N(5)**	-0.841	-0.726	-0.73	-0.744	-0.759	-0.757	-0.744	-0.710	-0.672
**N(6)**	-0.816	-0.723	-0.75	-0.721	-0.696	-0.696	-0.681	-0.572	-0.398
**N(7)**	-0.800	-0.765	-0.80	-0.777	-0.729	-0.728	-0.706	-0.544	-0.390
**N(8)**	-0.836	-0.779	-0.80	-0.783	-0.773	-0.773	-0.759	-0.744	-0.715
**C(1)**	0.495	0.442	0.39	0.469	0.557	0.558	0.574	0.502	0.415
**C(2)**	-0.011	-0.015	-0.01	-0.014	-0.028	-0.028	-0.030	-0.029	-0.030
**C(3)**	0.596	0.604	0.57	0.601	0.636	0.637	0.649	0.651	0.657
**C(4)**	0.543	0.582	0.48	0.551	0.630	0.630	0.641	0.633	0.632
**C(5)**	0.816	0.856	0.81	0.859	0.881	0.881	0.880	0.837	0.781
**C(6)**	0.837	0.852	0.83	0.868	0.883	0.883	0.883	0.833	0.777
**C(7)**	0.462	0.423	0.31	0.413	0.536	0.536	0.555	0.481	0.406
**O(1)**		-1.164			-	-1.081	-1.097		
**C**		1.658			-	2.149	1.807		
**O(2)**		-1.163			-	-1.083	-1.093		

Table S4b. B3LYP/6-311G* QTAIM charges of [**NiLH**] complexes.

Atoms	^{1}[NiLH]^{0}	^{1}[NiLH-CO$_2$]^{0}	^{2}[NiLH]$^{+}$	^{2}[NiLH-CO$_2$]$^{+}$	^{1}[NiLH]$^{2+}$	CO$_2$
**Ni**	0.151	0.675	0.663	0.890	0.944	
**S(1)**	-0.115	-0.099	-0.056	-0.034	0.013	
**S(2)**	-0.117	-0.065	-0.028	0.0002	0.056	
**N(1)**	-0.676	-0.676	-0.681	-0.689	-0.675	
**N(2)**	-0.660	-0.656	-0.669	-0.671	-0.665	
**N(3)**	-0.664	-0.667	-0.678	-0.687	-0.673	
**N(4)**	-0.681	-0.674	-0.666	-0.666	-0.664	
**N(5)**	-0.683	-0.688	-0.683	-0.686	-0.657	
**N(6)**	-0.664	-0.652	-0.655	-0.653	-0.644	
**N(7)**	-0.658	-0.679	-0.684	-0.695	-0.683	
**N(8)**	-0.684	-0.664	-0.663	-0.660	-0.650	
**C(1)**	0.319	0.331	0.315	0.313	0.303	
**C(2)**	-0.017	-0.027	-0.019	-0.020	-0.020	
**C(3)**	0.278	0.264	0.303	0.295	0.268	
**C(4)**	0.594	0.575	0.529	0.515	0.484	
**C(5)**	0.328	0.316	0.311	0.312	0.311	
**C(6)**	0.270	0.272	0.273	0.265	0.241	
**C(7)**	0.585	0.613	0.529	0.520	0.496	
**O(1)**		-1.214		-1.104		-1.076
**C**		1.442		1.749		2.153
**O(2)**		-1.205		-1.100		-1.076

Table S5a. B3LYP/6-311G* QTAIM BCP characteristics of [**NiL**] species from QTAIM analysis, i.e., A1-BCP-A2 distance *d*, charge density ρ, Laplacian Δρ, ellipticity ε and delocalization index DI of chosen complexes.

	**A1**	**A2**	***d*** **[Å]**	**ρ** **[e/Å^3]**	**Δρ** **[e/Å^5]**	**ε**	**λ_1** **[e/Å^5]**	**λ_2** **[e/Å^5]**	**λ_3** **[e/Å^5]**	**DI**
^{3}[**NiL**]$^{2-}$	Ni	N(1)	1.917	0.70	13.20	0.05	-3.09	-2.96	19.25	0.59
	Ni	N(3)	1.869	0.79	14.80	0.03	-3.63	-3.53	21.96	0.64
	Ni	N(5)	1.896	0.73	13.28	0.03	-3.24	-3.15	19.67	0.58
	Ni	N(7)	1.830	0.86	16.32	0.04	-4.09	-3.94	24.35	0.69
^{3}[**NiL-CO$_2$**]$^{2-}$	Ni	N(1)	1.991	0.59	9.88	0.08	-2.48	-2.29	14.65	0.48
	Ni	N(3)	2.051	0.52	8.69	0.08	-2.15	-1.99	12.83	0.42
	Ni	N(5)	1.953	0.65	11.20	0.03	-2.76	-2.67	16.63	0.50
	Ni	N(7)	1.957	0.66	11.19	0.02	-2.88	-2.83	16.90	0.52
	Ni	C	2.052	0.63	1.86	0.06	-3.13	-2.96	7.95	0.57
	C	O(1)	1.221	2.68	-4.33	0.02	-23.39	-23.03	42.09	1.26
	C	O(2)	1.222	2.67	-4.32	0.02	-23.31	-22.89	41.88	1.25
^{1}[**NiL**]$^{2-}$	Ni	N(1)	1.919	0.70	11.29	0.03	-3.03	-2.93	17.25	0.59
	Ni	N(3)	1.917	0.80	12.03	0.04	-3.76	-3.61	19.40	0.65
	Ni	N(5)	1.853	0.86	12.23	0.05	-4.21	-4.00	20.44	0.72
	Ni	N(7)	1.874	0.80	12.03	0.04	-3.76	-3.61	19.40	0.65
^{2}[**NiL**]$^{2-}$	Ni	N(1)	1.911	0.71	11.40	0.03	-3.13	-3.04	17.56	0.60
	Ni	N(3)	1.907	0.73	12.18	0.04	-3.25	-3.12	18.55	0.61
	Ni	N(5)	1.851	0.85	12.81	0.03	-4.05	-3.95	20.82	0.70
	Ni	N(7)	1.853	0.84	12.72	0.03	-3.98	-3.86	20.56	0.68
^{1}[**NiL**]^0	Ni	N(1)	1.901	0.73	11.66	0.03	-3.25	-3.15	18.06	0.61
	Ni	N(3)	1.910	0.72	12.04	0.02	-3.23	-3.16	18.42	0.60
	Ni	N(5)	1.834	0.88	13.37	0.03	-4.24	-4.12	21.73	0.69
	Ni	N(7)	1.862	0.82	13.01	0.01	-3.82	-3.79	20.62	0.67
^{1}[**NiL$_{fr}$CO$_2$**]^0	Ni	N(1)	1.893	0.79	11.28	0.02	-3.81	-3.75	18.84	0.62
	Ni	N(3)	1.959	0.67	9.99	0.04	-3.06	-2.96	16.01	0.53
	Ni	N(5)	1.840	0.89	12.27	0.06	-4.45	-4.19	20.91	0.67
	Ni	N(7)	1.894	0.77	11.19	0.07	-3.59	-3.36	18.13	0.60
	Ni	C	1.914	0.77	3.16	0.02	-4.01	-3.94	11.12	0.47
	C	O(1)	1.200	2.82	-3.19	0.00	-24.84	-24.74	46.38	1.29
	C	O(2)	1.201	2.81	-3.12	0.01	-24.76	-24.55	46.19	1.28
^{2}[**NiL**]$^+$	Ni	N(1)	1.913	0.71	11.43	0.02	-3.20	-3.12	17.75	0.58
	Ni	N(3)	1.836	0.88	13.59	0.02	-4.24	-4.14	21.96	0.68
	Ni	N(5)	1.906	0.74	11.92	0.02	-3.33	-3.27	18.52	0.60
	Ni	N(5)	1.864	0.82	13.13	0.01	-3.80	-3.77	20.70	0.66
^{1}[**NiL**]$^{2+}$	Ni	N(1)	1.919	0.70	11.29	0.03	-3.03	-2.93	17.25	0.59
	Ni	N(3)	1.905	0.80	12.03	0.04	-3.76	-3.61	19.40	0.65
	Ni	N(5)	1.842	0.80	12.03	0.04	-3.76	-3.61	19.40	0.65
	Ni	N(7)	1.872	0.86	12.23	0.05	-4.21	-4.00	20.44	0.72

Table S5b. B3LYP/6-311G* QTAIM BCP characteristics of [**NiLH**] species and CO_2 from QTAIM analysis, i.e., A1-BCP-A2 distance *d*, charge density ρ, Laplacian Δρ, ellipticity ε and delocalization index DI of chosen complexes.

	A1	A2	*d* [Å]	ρ [e/Å^3]	Δρ [e/Å^5]	ε	λ_1 [e/Å^5]	λ_2 [e/Å^5]	λ_3 [e/Å^5]	DI
^{1}[**NiLH**]$^{2+}$	Ni	N(1)	1.951	0.67	10.90	0.11	-2.90	-2.60	16.39	0.56
	Ni	N(3)	1.953	0.67	10.70	0.07	-2.91	-2.72	16.40	0.57
	Ni	N(5)	1.915	0.74	11.90	0.08	-3.36	-3.10	17.85	0.61
	Ni	N(7)	1.956	0.67	10.44	0.074	-2.89	-2.69	16.03	0.57
^{2}[**NiLH**]$^+$	Ni	N(1)	2.096	0.46	8.37	0.08	-1.64	-1.52	11.53	0.41
	Ni	N(3)	2.064	0.50	9.16	0.05	-1.88	-1.79	12.83	0.44
	Ni	N(5)	2.057	0.51	9.34	0.09	-1.89	-1.73	12.97	0.43
	Ni	N(7)	2.055	0.51	9.48	0.08	-1.87	-1.73	13.09	0.45
^{2}[**NiLH-CO$_2$**]$^+$	Ni	N(1)	2.111	0.46	7.51	0.11	-1.80	-1.62	10.94	0.38
	Ni	N(3)	2.086	0.49	8.10	0.08	-1.95	-1.80	11.85	0.41
	Ni	N(5)	2.060	0.52	8.69	0.07	-2.07	-1.94	12.70	0.42
	Ni	N(7)	2.084	0.50	8.20	0.06	-1.87	-1.77	11.84	0.42
	Ni	C	2.079	0.59	1.65	0.05	-2.85	-2.71	7.21	0.50
	C	O(1)	1.204	2.78	-3.35	0.01	-24.54	-24.25	45.44	1.31
	C	O(2)	1.205	2.78	-3.32	0.01	-24.54	-24.20	45.43	1.30
^{1}[**NiLH**]^0	Ni	N(1)	2.966	0.10	1.16	0.79	-0.23	-0.13	1.52	0.10
	Ni	N(3)	1.913	0.70	13.40	0.05	-3.10	-2.96	19.46	0.63
	Ni	N(5)	2.769	0.14	1.64	0.38	-040	-0.29	2.33	0.13
	Ni	N(7)	1.888	0.74	14.12	0.00	-3.39	-3.38	20.88	0.66
^{1}[**NiLH-CO$_2$**]^0	Ni	N(1)	3.086	0.08	0.93	0.33	-0.17	-0.13	1.23	0.06
	Ni	N(3)	2.006	0.57	10.16	0.04	-2.30	-2.22	14.68	0.49
	Ni	N(5)	2.219	0.39	5.16	0.17	-1.66	-1.42	8.24	0.30
	Ni	N(7)	2.008	0.57	10.03	0.07	-2.34	-2.20	14.56	0.49
	Ni	C	1.888	0.93	3.50	0.14	-5.21	-4.56	13.27	0.86
	C	O(1)	1.251	2.50	-6.96	0.03	-21.56	-20.87	35.46	1.17
	C	O(2)	1.254	2.48	-6.91	0.03	-21.22	-20.52	34.83	1.17
**CO$_2$**	C	O(1)	1.200	2.82	-3.19	0.00	-24.84	-24.74	46.38	1.29
	C	O(2)	1.201	2.82	-3.19	0.00	-24.84	-24.74	46.38	1.29

Table S6a. B3LYP/6-311G* Mulliken populations of [**NiL**] species for localized molecular orbitals on the nickel atom for complexes studied.

	s	d_{z2}	d_{xz}	d_{yz}	$d_{x^2-y^2}$	d_{xy}	Σd+s
^{3}[**NiL**]$^{2-}$α	0.23	0.81	-	-	-	-	1.04
	-	-	0.21	0.43	0.24	-	0.85
	-	-	-	0.16	0.73	-	0.99
	-	-	-	0.58	0.33	-	0.91
	-	-	-	-	-	0.87	0.87
^{3}[**NiL**]$^{2-}$β	0.33	0.75	-	-	-	-	1.08
	-	-	0.62	0.27	-	-	0.89
	-	-	0.24	0.62	-	-	0.86
	-	-	0.07		-	0.86	0.93
^{1}[**NiL**]$^{2-}$	0.21	0.84					1.05
			0.78	0.17			0.97
			0.18	0.72			0.94
						0.90	0.90
^{2}[**NiL**]$^-$α	0.19	0.86					1.05
			0.73	0.23			0.96
			0.21	0.71			0.92
						0.94	0.94
^{2}[**NiL**]$^{2-}$β	0.19	0.86					1.05
			0.72	0.24			0.96
			0.23	0.71			0.94
						0.95	0.95
^{1}[**NiL**]^0	0.17	0.87	-	-	-	-	1.04
	-	-	0.64	0.33	-	-	0.97
	-	-	0.31	0.62	-	-	0.93
	-	-	-	-	-	0.95	0.95
^{1}[**NiL**]$^{2+}$	0.13	0.89					1.03
			0.92				0.94
			0.00	0.92			0.95
						0.93	0.96
^{2}[**NiL**]$^+$α	0.15	0.88					1.03
		0.59	0.33				0.93
			0.35	0.61			0.97
						0.95	0.96
^{2}[**NiL**]$^+$β	0.15	0.88					1.03
				0.94			0.94
			0.94				0.94
						0.95	0.95
^{1}[**NiL-CO$_2$**]^0	0.17	0.87					1.04
	-	-	0.64	0.33	-	-	0.97
	-	-	0.31	0.62	-	-	0.93
	-	-	-	-	-	0.95	0.95
^{1}[**NiL$_{fr}$CO$_2$**]^0	-	-	0.84	0.11	-	-	0.95
	-	-	0.12	0.86	-	-	0.98
	-	-	-	-	-	0.96	0.96
^{3}[**NiL-CO$_2$**]$^{2-}$α	-	0.74	-	-	-	-	0.93
	-	-	0.48	0.4	-	-	0.88
	-	-	0.43	0.54	-	-	0.97
	-	-	-	-	0.92	-	0.92
	-	-	-	-	-	0.93	0.74
^{3}[**NiL-CO$_2$**]$^{2-}$β	-	-	0.51	0.46	-	-	0.97
	-	-	0.44	0.49	-	-	0.93
	-	-	-	-	-	0.94	0.94

Table S6b. B3LYP/6-311G* Mulliken populations of [**NiLH**] species for localized molecular orbitals on the nickel atom for complexes studied.

	s	d_{z2}	d_{xz}	d_{yz}	$d_{x^2-y^2}$	d_{xy}	Σd+s
^{1}[**NiLH**]$^{2+}\alpha$	0.16	0.87	0.01				1.06
		0.01	0. 93	0.03			0.97
			0.03	0.71		0.23	0.97
			0.01	0.23	0.41	0.73	0.96
^{1}[**NiLH**]$^{2+}\beta$	0.19	0.87					1.06
			0.94	0.03			0.97
				0.22		0.74	0.96
			0.02	0.72		0.22	0.97
^{2}[**NiLH**]$^{+}\alpha$	0.23	0.81	0.03				1.07
				0.01	0.98		1.00
		0.03	0.84	0.05		0.06	0.96
				0.65	0.01	0.29	0.96
			0.09	0.25		0.60	0.95
^{2}[**NiLH**]$^{+}\beta$	0.35	0.74	0.03				1.12
		0.03	0.81	0.07		0.06	0.96
				0.58		0.36	0.95
			0.11	0.30		0.53	0.94
^{2}[**NiLH-CO$_2$**]$^{+}\alpha$					1.00		1.00
		0.01	0.46	0.49			0.98
			0.50	0.46			0.96
		0.02		0.01		0.94	0.97
	0.09	0.82				0.02	0.93
^{2}[**NiLH-CO$_2$**]$^{+}\beta$			0.49	0.47			0.97
			0.47	0.49			0.96
						0.95	0.96
^{1}[**NiLH**]^{0}	0.60	0.45	0.02		0.08		1.15
			0.22			0.68	0.91
			0.66	0.03	0.02	0.22	0.93
	0.02	0.08		0.54	0.29		0.94
	0.02	0.24	0.02	0.36	0.30		0.94
^{1}[**NiLH-CO$_2$**]^{0}	0.16	0.10		0.22	0.55		1.03
		0.07	0.73	0.12		0.04	0.96
		0.09	0.15	0.55	0.16		0.95
	0.05					0.89	0.94

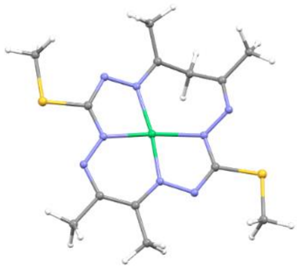	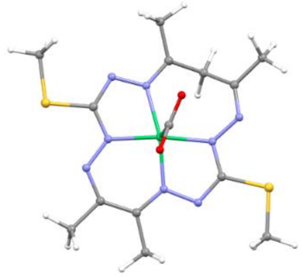
^{3}[**NiL**]$^{2-}$	^{3}[**NiL-CO$_2$**]$^{2-}$
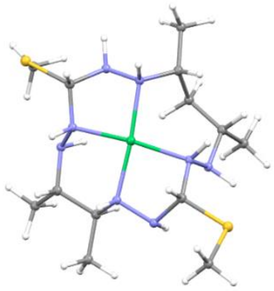	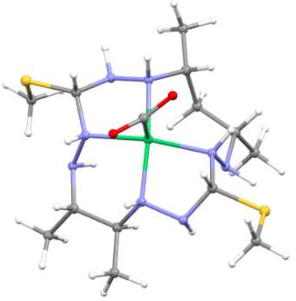
^{2}[**NiLH**]$^{+}$	^{2}[**NiLH-CO$_2$**]$^{+}$
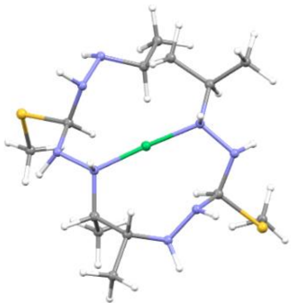	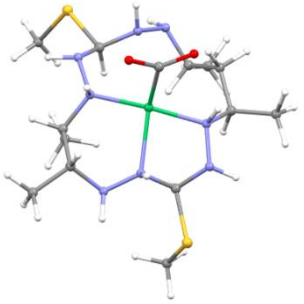
^{1}[**NiLH**]^{0}	^{1}[**NiLH-CO$_2$**]^{0}

Figure S1. Comparison of geometries of complexes studied without (left column) and with CO_2 (right column).

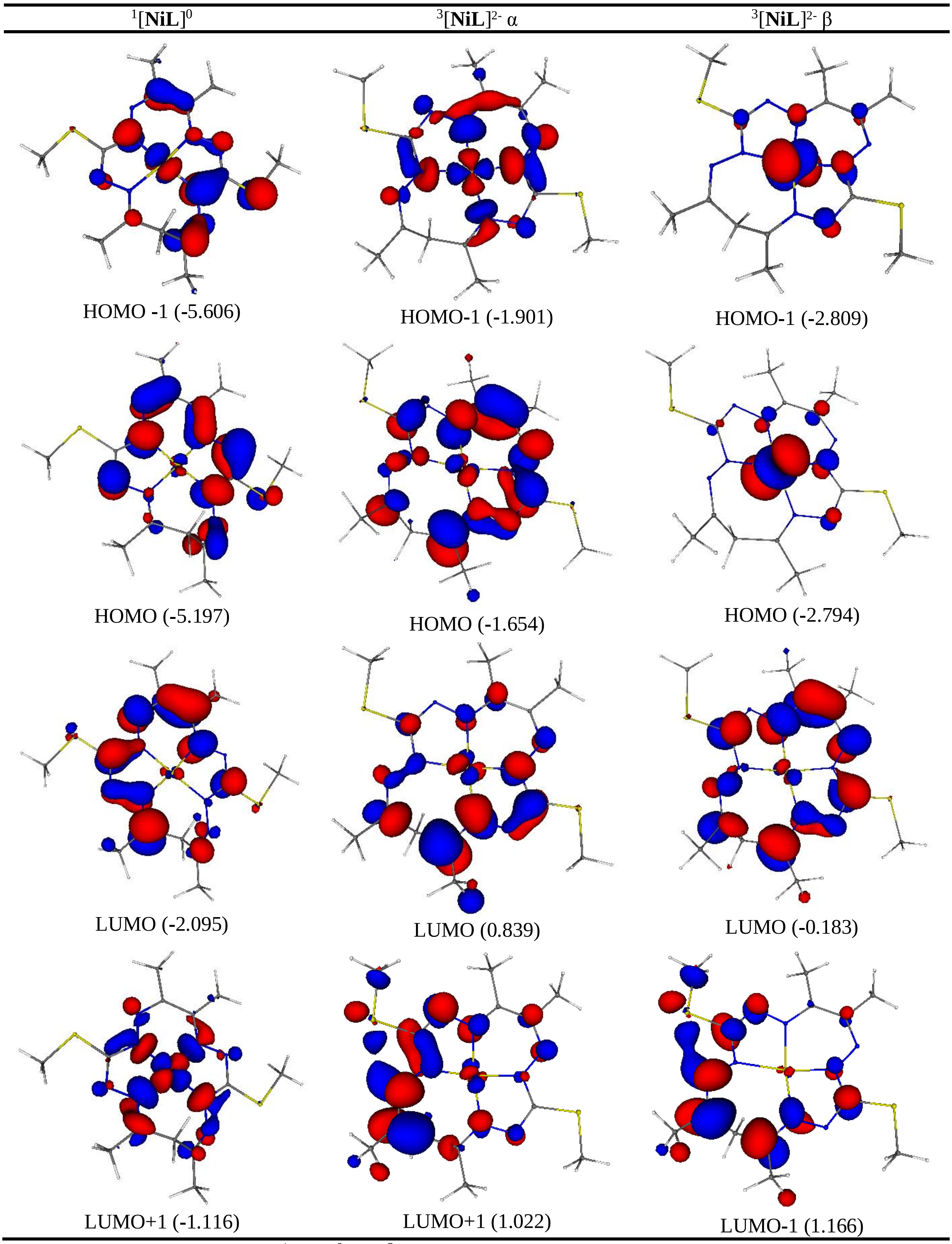

Figure S2a. Frontier orbitals of ^{1}[**NiL**]^{0} and ^{3}[**NiL**]$^{2-}$ (eigenvalues are shown in parentheses in eV), isosurface value is 0.04 e bohr^{-3}.

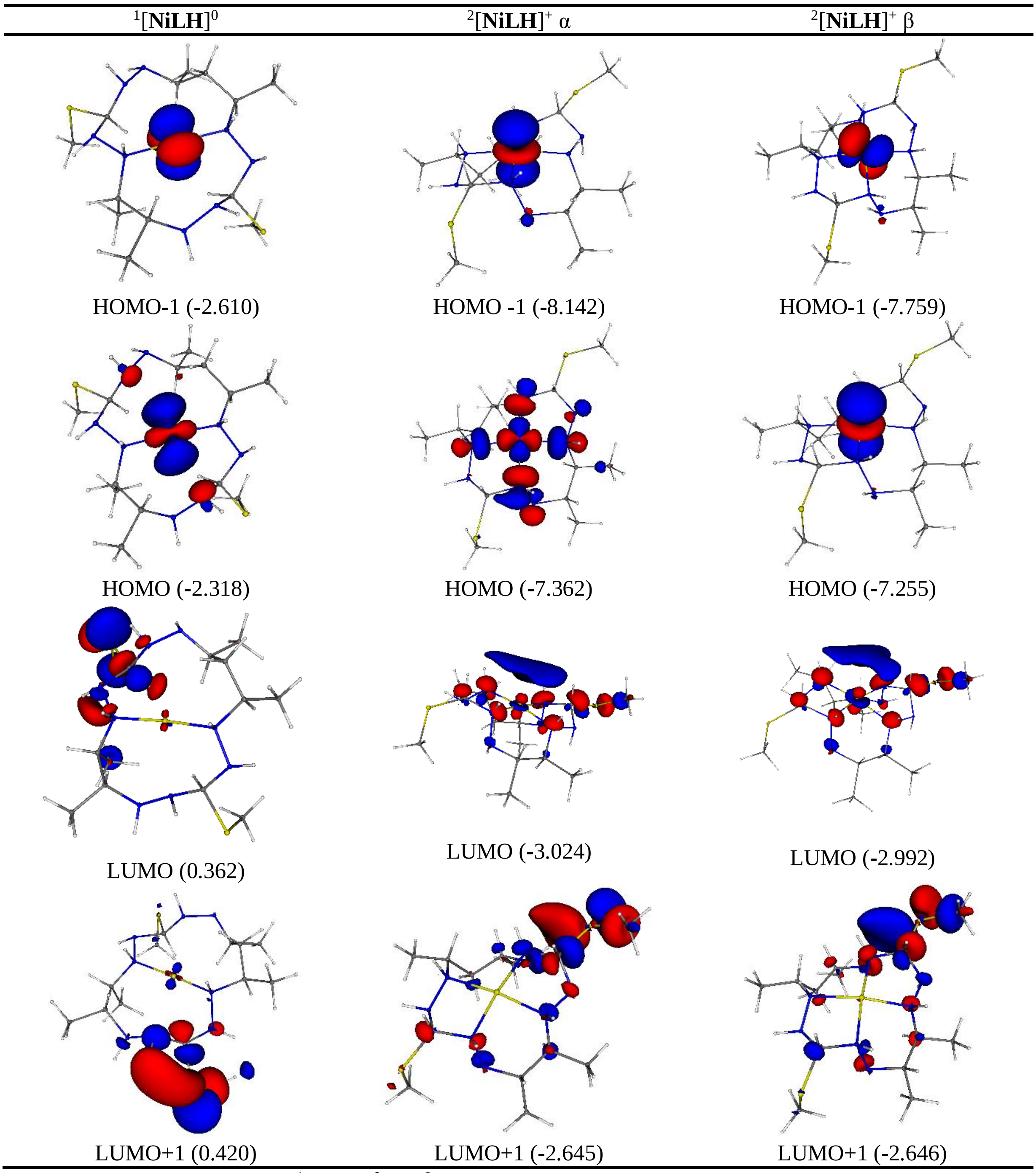

Figure S2b. Frontier orbitals of ^{1}[**NiLH**]^{0} and ^{2}[**NiLH**]$^{+}$ (eigenvalues are shown in parentheses in eV), isosurface value is 0.04 e bohr^{-3}.

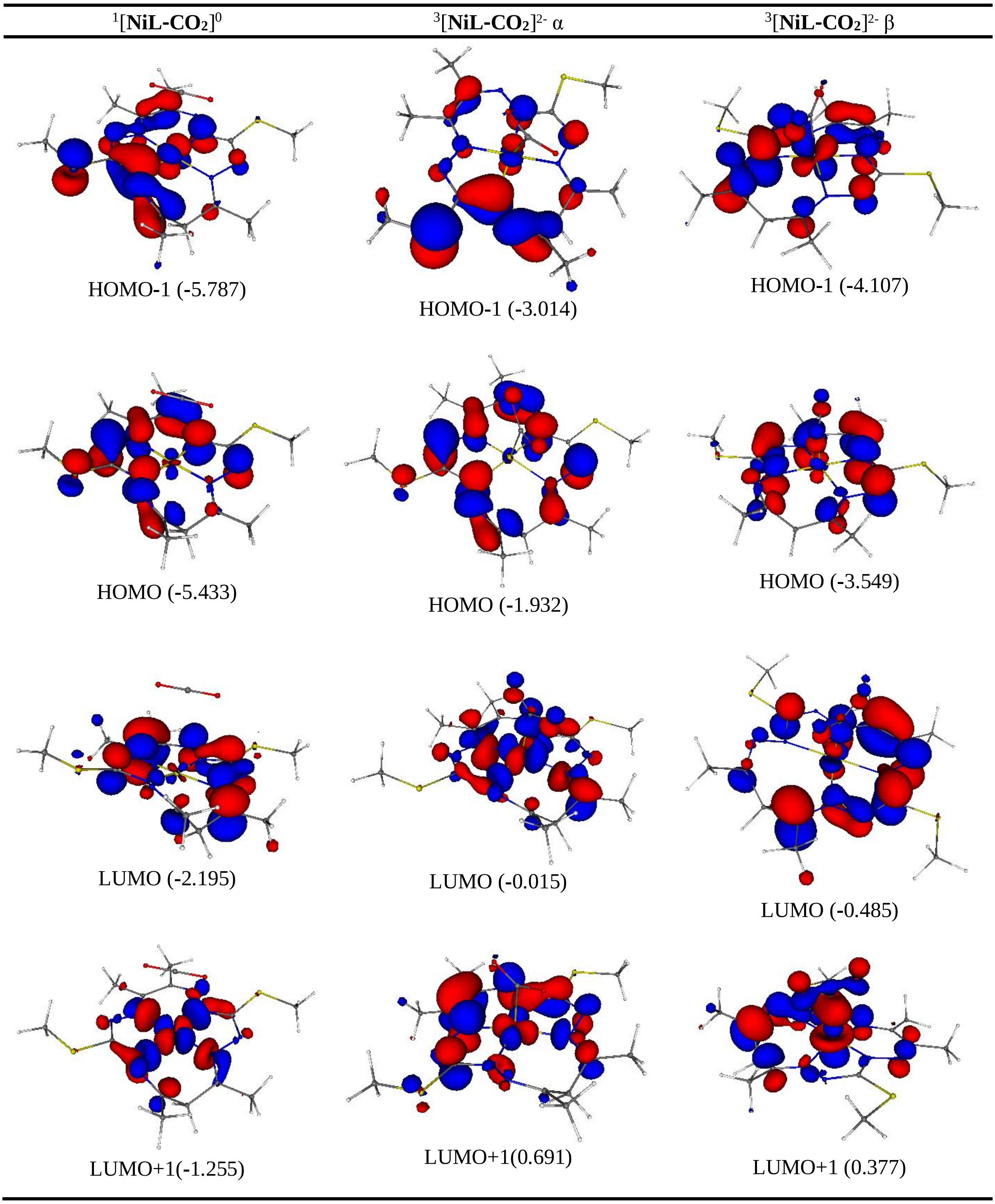

Figure S3a. Frontier orbitals of ^{1}[**NiL-CO$_2$**]^0 and ^{3}[**NiL-CO$_2$**]$^{2-}$ (eigenvalues are shown in parentheses in eV), isosurface value is 0.04 e bohr^{-3}.

^{1}[**NiLH-CO$_2$**]^{0}	^{2}[**NiLH-CO$_2$**]$^{+}$ α	^{2}[**NiLH-CO$_2$**]$^{+}$ β
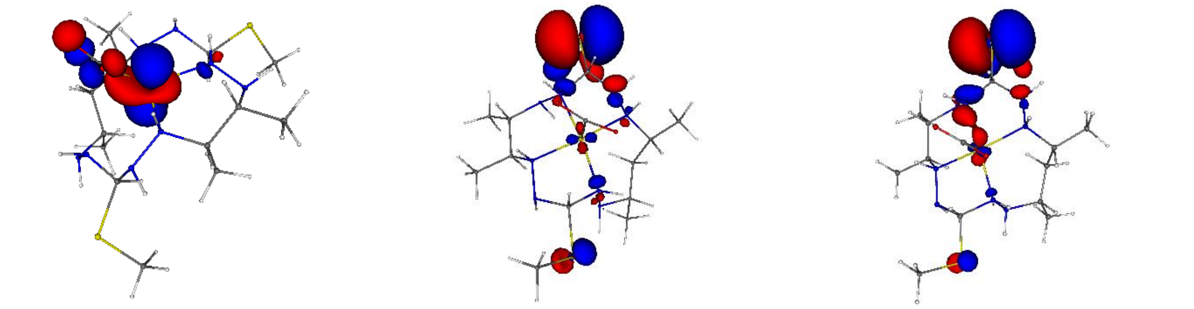		
HOMO-1 (-4.618)	HOMO-1 (-9.389)	HOMO-1 (-9.346)
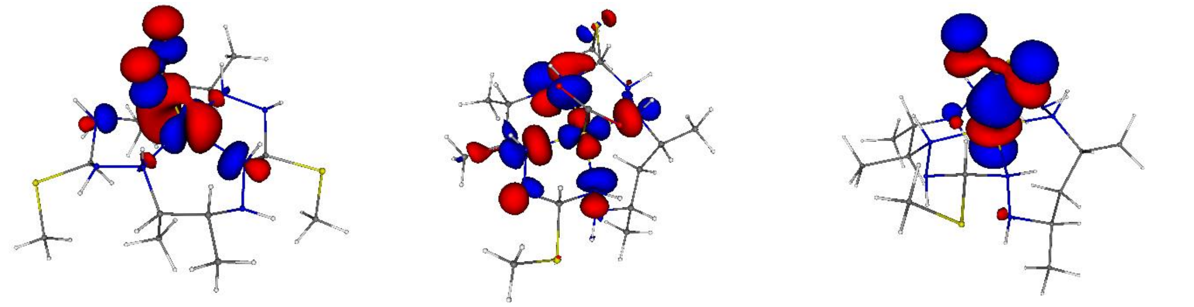		
HOMO (-3.722)	HOMO (-8.459)	HOMO (-8.920)
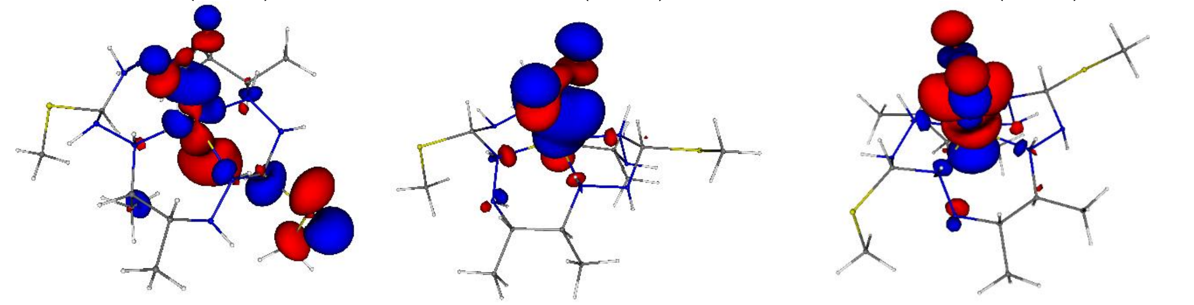		
LUMO (-0.339)	LUMO (-4.502)	LUMO (-4.412)
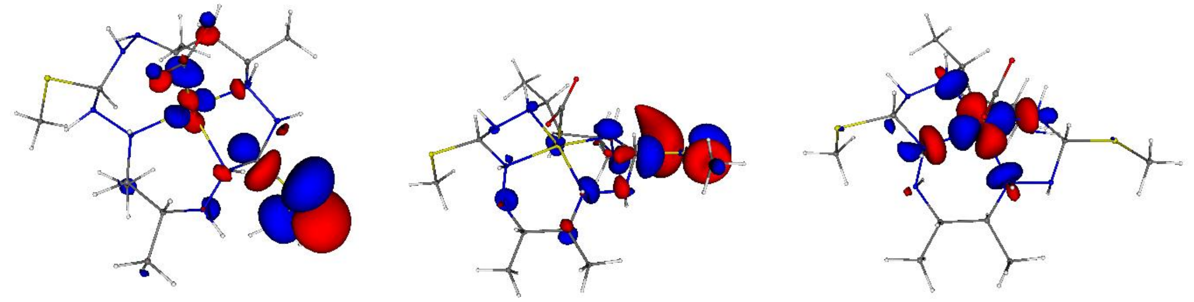		
LUMO+1 (-0.061)	LUMO+1 (-2.926)	LUMO+1 (-3.309)

Figure S3b. Frontier orbitals of ^{1}[**NiLH-CO$_2$**]^{0} and ^{2}[**NiLH-CO$_2$**]$^{+}$ (eigenvalues are shown in parentheses in eV), isosurface value is 0.04 e bohr^{-3}.
