# Supplementary material for: Ni Oxidation State and Ligand Saturation Impact on the Capability of Octaazamacrocyclic Complexes to Bind and Reduce CO2
Source: Molecules. 2021 Jul 7;26(14):4139. doi: 10.3390/molecules26144139 (PMC8307626; doi:10.3390/molecules26144139)
Supplement: Supplementary file 1 [file molecules-26-04139-s001.zip › SI_XYZ.zip!/XYZ_compounds.pdf]

Table SI_XYZ. Total B3LYP/6-311G* energies/enthalpies of different species studied including the xyz file names contained in this SI_XYZ.zip.

	E	H	NAME.xyz
^{3}[**NiL**]$^{2-}$	-3250.503603	-3250.142369	3NiL2_Red.xyz
^{1}[**NiL**]$^{2-}$	-3250.510626	-3250.118574	1NiL2_Red.xyz
^{2}[**NiL**]$^{-}$	-3250.466015	-3250.101912	2NiL1_Red.xyz
^{1}[**NiL**]^{0}	-3250.378540	-3250.011645	1NiL0.xyz
^{2}[**NiL**]$^{+}$	-3250.186230	-3249.819337	2NiL1_Ox.xyz
^{1}[**NiL**]$^{2+}$	-3249.948182	-3249.580637	1NiL2_Ox.xyz
^{1}[**NiLH**]^{0}	-3258.616497	-3258.079627	1NiLH0.xyz
^{2}[**NiLH**]$^{+}$	-3258.578456	-3258.036079	2NiLH1.xyz
^{2}[**NiLH$_{C1}$**]$^{+}$	-3258.574194	-3258.031725	2NiLH1_C1.xyz
^{2}[**NiLH$_{C5}$**]$^{+}$	-3258.577090	-3258.034923	2NiLH1_C5.xyz
^{1}[**NiLH**]$^{2+}$	-3258.432714	-3257.886345	1NiLH2.xyz
^{1}[**NiLH$_{C1}$**]$^{2+}$	-3258.428449	-3257.881841	1NiLH2_C1.xyz
^{1}[**NiLH$_{C3}$**]$^{2+}$	-3258.430081	-3257.883263	1NiLH2_C3.xyz
^{1}[**NiLH$_{C5}$**]$^{2+}$	-3258.430207	-3257.882894	1NiLH2_C5.xyz
^{1}[**NiLH$_{C1C3}$**]$^{2+}$	-3258.422065	-3257.875684	1NiLH2_C1_C3.xyz
^{3}[**NiL-CO$_2$**]$^{2-}$	-3439.157621	-3438.780621	3NiLCO22_Red.xyz
^{1}[**NiL-CO$_2$**]$^{2-}$	-3439.157234	-3438.778617	1NiLCO22_Red.xyz
^{2}[**NiL-CO$_2$**]$^{-}$	-3439.111696	-3438.730981	2NiLCO21_Red.xyz
^{1}[**NiL-CO$_2$**]^{0}	-3439.023880	-3438.640248	1NiLCO20.xyz
^{2}[**NiL-CO$_2$**]$^{+}$	-3438.830920	-3438.448238	2NiLCO21_Ox.xyz
^{1}[**NiL-CO$_2$**]$^{2+}$	-3438.592560	-3438.208037	1NiLCO22_Ox.xyz
^{1}[**NiLH-CO$_2$**]^{0}	-3447.321361	-3446.765337	1NiLHCO20.xyz
^{2}[**NiLH-CO$_2$**]$^{+}$	-3447.22514	-3446.665791	2NiLHCO21.xyz
^{2}[**NiLH$_{C1}$-CO$_2$**]$^{+}$	-3447.221017	-3446.661914	2NiLHCO21_C1.xyz
^{2}[**NiLH$_{C5}$-CO$_2$**]$^{+}$	-3447.224161	-3446.664701	2NiLHCO21_C5.xyz
^{1}[**NiL$_{fr}$CO$_2$**]^{0}	-3438.976674	-3438.542170	1NiLfrCO20.xyz
**CO_2**	-188.643673	-188.628479	CO2.xyz
